# Prediction of fluid responsiveness: an update

**DOI:** 10.1186/s13613-016-0216-7

**Published:** 2016-11-17

**Authors:** Xavier Monnet, Paul E. Marik, Jean-Louis Teboul

**Affiliations:** 1 Medical Intensive Care Unit, Bicêtre Hospital, Paris-Sud University Hospitals, Inserm UMR_S999, Paris-Sud University, 78, rue du Général Leclerc, 94 270 Le Kremlin-Bicêtre, France; 2 Department of Medicine, Division of Pulmonary and Critical Care Medicine, Eastern Virginia Medical School, Norfolk, VA USA

**Keywords:** Pulse pressure variation, Stroke volume variation, Pulse contour analysis, Heart–lung interactions, Passive leg raising, Fluid responsiveness, Cardiac preload, Stroke volume, Volume expansion, Fluid therapy, Haemodynamic monitoring, Critical care, Echocardiography, ICU, Operating room

## Abstract

In patients with acute circulatory failure, the decision to give fluids or not should not be taken lightly. The risk of overzealous fluid administration has been clearly established. Moreover, volume expansion does not always increase cardiac output as one expects. Thus, after the very initial phase and/or if fluid losses are not obvious, predicting fluid responsiveness should be the first step of fluid strategy. For this purpose, the central venous pressure as well as other “static” markers of preload has been used for decades, but they are not reliable. Robust evidence suggests that this traditional use should be abandoned. Over the last 15 years, a number of dynamic tests have been developed. These tests are based on the principle of inducing short-term changes in cardiac preload, using heart–lung interactions, the passive leg raise or by the infusion of small volumes of fluid, and to observe the resulting effect on cardiac output. Pulse pressure and stroke volume variations were first developed, but they are reliable only under strict conditions. The variations in vena caval diameters share many limitations of pulse pressure variations. The passive leg-raising test is now supported by solid evidence and is more frequently used. More recently, the end-expiratory occlusion test has been described, which is easily performed in ventilated patients. Unlike the traditional fluid challenge, these dynamic tests do not lead to fluid overload. The dynamic tests are complementary, and clinicians should choose between them based on the status of the patient and the cardiac output monitoring technique. Several methods and tests are currently available to identify preload responsiveness. All have some limitations, but they are frequently complementary. Along with elements indicating the risk of fluid administration, they should help clinicians to take the decision to administer fluids or not in a reasoned way.

## Background

Volume expansion, the first-line treatment of acute circulatory failure, can be the source of a crucial therapeutic dilemma. On the one hand, the severity of the disease incites one to initiate treatment rapidly and massively. In line with this, the pivotal study by Rivers et al. [1] showed that massive fluid administration during the first 6 h of resuscitation of patients with severe sepsis and septic shock was associated with improved outcome. On the other hand, it has now been clearly demonstrated that fluid overload has detrimental consequences. Fluid overload prolongs mechanical ventilation and increases the mortality of critically ill patients in general and, more specifically, in patients with sepsis [2–4], acute respiratory distress syndrome (ARDS) [5–7], intra-abdominal hypertension [8] and acute kidney injury [9, 10]. The potential benefit of volume expansion, related to an increase in cardiac output and oxygen delivery, must be balanced by the risk of aggravating lung and tissue oedema [11].

**Fig. 1:**
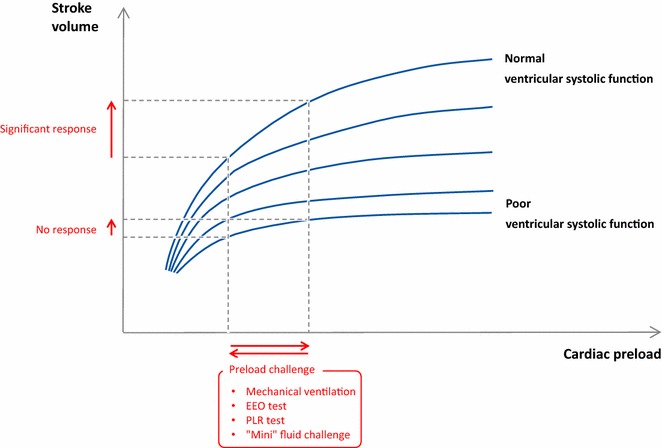
Frank–Starling relationship. The slope of the Frank–Starling curve depends on the ventricular systolic function. Then, one given level of cardiac preload does not help in predicting fluid responsiveness. By contrast, dynamic tests include a preload challenge (either spontaneous, induced by mechanical ventilation or provoked, by passive leg raising, end-expiratory occlusion or fluid infusion). Observing the resulting effects on stroke volume allows for the detection of preload responsiveness. *EEO* end-expiratory occlusion, *PLR* passive leg raising

The response to a fluid challenge is complicated by cardiovascular physiology [12]. Due to varying shapes that the Frank–Starling curve could take depending on the ventricular systolic function, a fluid challenge could lead to either a significant or a negligible increase in stroke volume and cardiac output (Fig. 1). If no attempts are made to predict the response of cardiac output to volume expansion, “fluid responsiveness” occurs in only half the patients [13]. Should volume expansion fail to result in a significant haemodynamic improvement, it inherently leads to haemodilution, to increased cardiac filling pressures and eventually to fluid overload. All these facts taken together lead one to view fluid therapy as any other medication, which must be neither overdosed nor under-dosed. Moreover, it argues for a careful prediction of the effects of fluids before they are administered when these effects are not sure, i.e. after the very initial phase of circulatory failure and/or if fluid losses are not obvious. For this prediction, the method that has been used for decades, namely central venous pressure (CVP), has been demonstrated to be unreliable. Conversely, a number of “dynamic” methods have been developed to test preload responsiveness [14, 15]. In this review, we will summarise the most recent findings regarding this strategy of fluid management.

## Central venous pressure and static markers of cardiac preload: please, stop using it… for predicting fluid responsiveness!

The question of predicting fluid responsiveness with CVP use is quite controversial and somewhat perplexing. On the one hand, there is a tremendous amount of evidence that a given value of CVP does not predict fluid responsiveness. This has been established by a number of studies and meta-analyses [14, 16]. On the other hand, surveys regularly report that clinicians still continue to use CVP for predicting fluid responsiveness. The FENICE study, an observational study conducted in intensive care units (ICUs) around the World, showed that static markers of preload are still used to test preload responsiveness in one-third of instances [17]. In a survey regarding haemodynamic monitoring in patients undergoing high-risk surgery, 73% of American and 84% of European anaesthesiologists reported that they used the CVP to guide fluid management [18].

This inconsistency is even more difficult to understand since the inability of CVP to reflect preload responsiveness comes from simple physiology. A static value of CVP could correspond to preload responsiveness as well as preload unresponsiveness, depending on the shape of the Frank–Starling curve, which varies from one patient to another and, in a patient, from one time to another (Fig. 1). This is true even for relatively low CVP values [19]. What is true for the CVP is true for all static indicators of cardiac preload, such as the pulmonary artery occlusion pressure, the global end-diastolic volume measured with transpulmonary thermodilution and the flow time of aortic flow by oesophageal Doppler. It is also the case for the left ventricular end-diastolic dimensions measured by echocardiography—even if, otherwise, this method has the advantage to provide a full investigation of cardiac function and structure. The fact that CVP is unhelpful to assess preload responsiveness does not mean that it should not be measured in patients with or at risk of acute circulatory failure. The CVP is a good marker of preload (not preload responsiveness) and a key determinant of cardiac function. It is also one of the determinants of the pressure gradient for organ perfusion (mean arterial pressure minus CVP). High CVP values, because they impair renal perfusion, are associated with acute kidney injury [20, 21].

## Pulse pressure and stroke volume variation: well-established accurate indices…

The variations of stroke volume (SVV), and of surrogates, that are induced by mechanical ventilation were the first methods to be developed for the dynamic assessment of preload responsiveness. The rationale is that, during positive pressure ventilation, insufflation decreases preload of the right ventricle. When transmitted to the left side, this induces a decrease in preload of the left ventricle. If left ventricular stroke volume changes in response to cyclic positive pressure ventilation, this indicates that both ventricles are preload dependent. Mechanical ventilation can be used as a provocative test to challenge the slope of the Frank–Starling curve at the bedside (Fig. 1). The amplitude of arterial pulse pressure (the difference between systolic and diastolic pressures) during mechanical ventilation was first used to estimate stroke volume. In 2000, pulse pressure variation (PPV) was shown to predict the response of cardiac output to volume expansion [22]. This has been confirmed by several studies. To date, PPV is the marker of preload responsiveness that has accumulated the largest amount of evidence [14, 23, 24]. A recent meta-analysis which included 22 studies and 807 patients reported a pooled sensitivity for predicting fluid responsiveness of 88% with a specificity of 89%. The median threshold of the PPV was 12% (interquartile range 10–13%) [23] (Table 1).

**Table 1:** Summary of methods predicting preload responsiveness with diagnostic threshold and limitations

Method	Threshold	Main limitations
Pulse pressure/stroke volume variations [22]	12%	Cannot be used in case of spontaneous breathing, cardiac arrhythmias, low tidal volume/lung compliance
Inferior vena cava diameter variations [44]	12%	Cannot be used in case of spontaneous breathing, low tidal volume/lung compliance
Superior vena caval diameter variations [44]	36%*	Requires performing transesophageal Doppler Cannot be used in case of spontaneous breathing, low tidal volume/lung compliance
Passive leg raising [55]	10%	Requires a direct measurement of cardiac output
End-expiratory occlusion test [75]	5%	Cannot be used in non-intubated patients Cannot be used in patients who interrupt a 15-s respiratory hold
“Mini”-fluid challenge (100 mL) [84]	6%**	Requires a precise technique for measuring cardiac output
“Conventional” fluid challenge (500 mL) [81]	15%	Requires a direct measurement of cardiac output Induces fluid overload if repeated

* Thresholds from 12 to 40% have been reported

** 10% is more compatible with echography precision. Citations indicate the most important reference regarding the test

The diagnostic accuracy of PPV has been analysed through the prism of the “grey zone analysis”. Using complex statistical methods, a study demonstrated that there is a grey zone of PPV values, between 9 and 13%, where the sensitivity or the specificity is lower than 90% [25]. It was estimated that 24% of PPV values encountered in practice remain within these limits [25]. The concept of the grey zone analysis has been widely adopted, and some have used it to question the validity of the PPV. Nevertheless, the grey zone analysis only expresses the fact that, as for any continuous diagnostic variable, the farther PPV from the diagnostic threshold, the stronger the accuracy of the prediction of fluid responsiveness or unresponsiveness.

Following invasive arterial pulse pressure, many other surrogates of stroke volume have been investigated to assess SVV during mechanical ventilation. In recent years, research has focused on less-invasive and non-invasive techniques. These techniques may be particularly useful when no arterial line is in place, typically in the operating room. The ventilation-induced variations in arterial pulse pressure estimated by volume-clamp photoplethysmography [26], stroke volume measured by pulse contour analysis, the velocity time integral of the flow in the left ventricular outflow track at echocardiography, the aortic blood flow by oesophageal Doppler [27], and the amplitude of the plethysmographic signal [28, 29] have been established as preload responsiveness indicators [23, 24]. The reliability of the latter index is likely lower in the ICU patients than in the operating room patients [30] and in case of vasopressors administration [31, 32]. It has even been suggested that the variations of the peak velocity in the carotid [33] or brachial [34] arteries could reflect PPV and detect preload responsiveness.

## … And some well-established limitations

While the utility of the PPV and SVV has become better established, those conditions in which they become unreliable have been more clearly defined. These are summarised in Table 2. The major conditions where the PPV and SVV are unreliable include spontaneous breathing (even in an intubated patient) and cardiac arrhythmias which result in false positive, and ARDS which result in false-negative outcomes (Table 2). In the case of ARDS, the low tidal volume, which is commonly used, reduces the amplitude of the change in intrathoracic pressure that causes the PPV and SVV. A recent study showed that in patients with ARDS, the tidal volume could be transiently increased to 8 mL/kg. If this “tidal volume challenge” results in an increase in the absolute value of PPV ≥ 3.5% or of SVV ≥ 2.5%, fluid responsiveness is very likely [35]. Importantly in patients with ARDS, not only the tidal volume, but also the low lung compliance prevents use of PPV and SVV since it reduces the transmission of alveolar pressures to intravascular and cardiac pressures [36]. It would appear that the poor diagnostic value of PPV in patients with ARDS is more closely related to the low lung compliance than to the low tidal volume [36, 37]. Intra-abdominal hypertension is also well recognised as another condition that limits the accuracy of PPV and SVV [38] (Table 2). In this case, respiratory variations of stroke volume are not exclusively related to volaemia [39], and threshold values identifying responders and non-responders might be higher than under normal intra-abdominal pressure [40]. Finally, it has been suggested that in case of right heart failure, the increase in right ventricular afterload during mechanical insufflation could be responsible for some false positives in PPV or SVV. Nevertheless, this has been poorly documented. A study suggesting this limitation reported a surprisingly high incidence of false positives that has never been reported in many studies investigating PPV or SVV, even in ARDS patients [41].

**Table 2:** Conditions where pulse pressure and stroke volume variations are less reliable

Spontaneous breathing	False+
Cardiac arrhythmias	False+
Low Vt/low lung compliance	False−
Open chest	False−
Increased intra-abdominal pressure	False+
Very high respiratory rate (HR/RR < 3.6)	False−
Right heart failure*	False+

* See text for details

In practice, the conditions where the reliability of PPV and SVV is decreased are quite common in the ICU. This is particularly true today since patients are less sedated and low tidal volume ventilation is more common than before and since cardiac arrhythmias are not uncommon. A recent prospective study reported an incidence of 17% of instances where the reliability of PPV and SVV could be used without limitation [42].

In the operating room setting, PPV and SVV monitoring (invasively or non-invasively obtained) retain their predictive value since the conditions of their applicability are generally fulfilled. The limitations of PPV and SVV must always be kept in mind by the intensivists or anaesthesiologists, since ignoring them could lead to serious misinterpretations. However, a recent survey demonstrated that a large proportion of intensivists did not have full knowledge of all factors confounding PPV and SVV interpretation [43].

## Variations of vena caval dimensions

The principle behind these indices is also based on heart–lung interactions, but they do not operate in the same way as PPV. The changes in intrathoracic pressure induced by mechanical ventilation may induce some variations in the venae cavae in close proximity to the heart when the central blood volume is low. The variation of the inferior vena cava diameter measured by transthoracic echocardiography has been reported to detect preload responsiveness with reasonable accuracy. The “collapsibility” of the superior vena cava has also been shown to reflect fluid responsiveness; however, it requires transesophageal echocardiography [15]. It has been recently reported that the detection of preload responsiveness was better with the respiratory variation of the superior vena cava diameter than with that of the inferior vena cava diameter [44].

Compared to PPV and SVV, the vena caval indices have been less well studied and the diagnostic thresholds that were reported have varied from 12% [45] to 40% [46] (Table 1). In a 2014 meta-analysis which included 8 studies, the pooled sensitivity was only 76% only and the pooled specificity was 86% [47]. In a more recent study in septic critically ill patients, the accuracy of ventilation-induced changes in inferior and superior vena cava diameter in predicting fluid responsiveness was found to be poor [48].

**Fig. 2:**
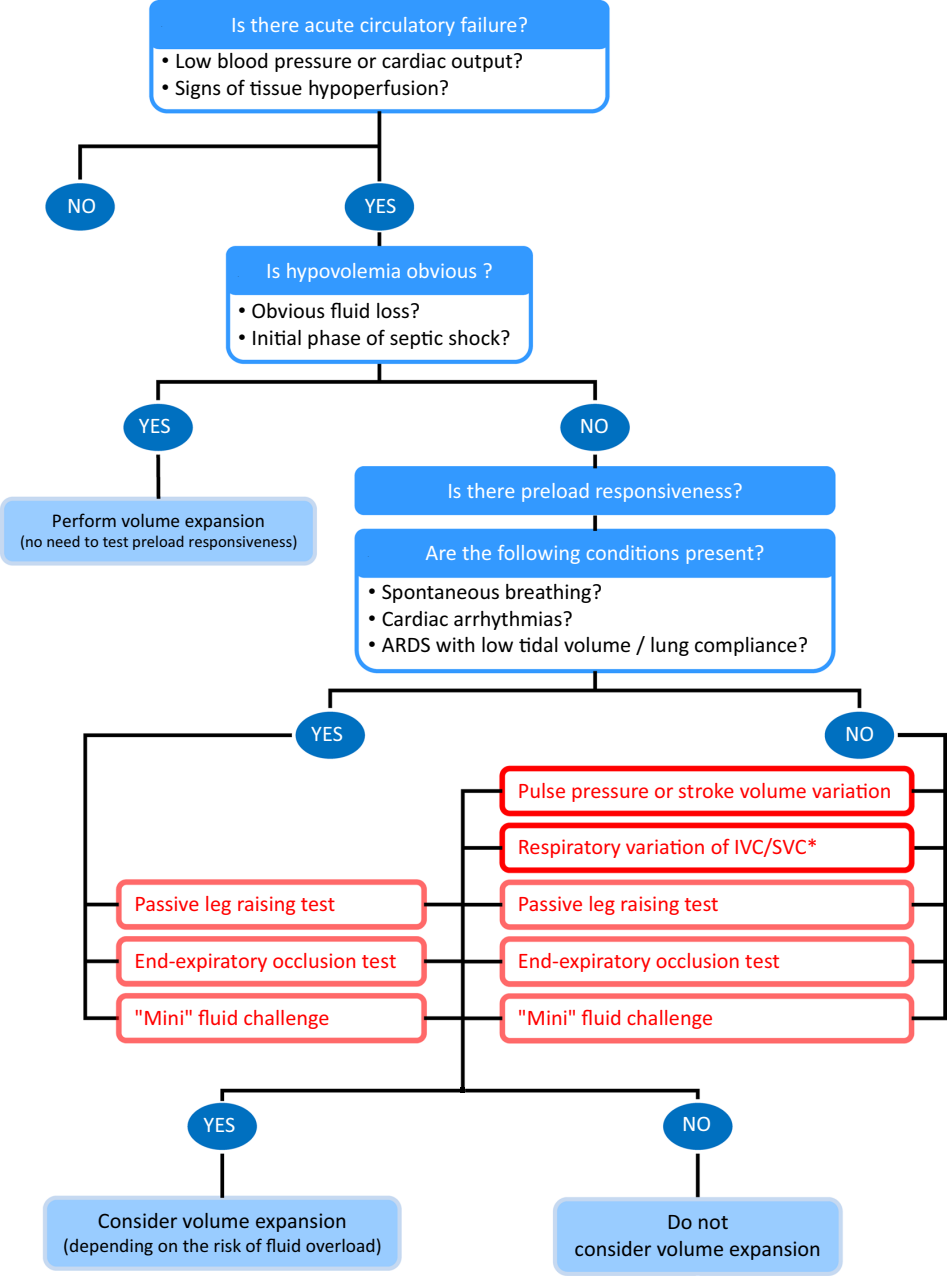
Fluid strategy.*The variation in inferior/superior vena cava diameters can be used in case of cardiac arrhythmias. *ARDS* acute respiratory distress syndrome, *IVC* inferior vena cava, *PCO*_*2*_ *gap* veno-arterial difference in carbon dioxide tension, *SVC* superior vena cava

It is important to take into account that the variation of vena caval diameter shares many of the same limitations as with PPV (Fig. 2). As anticipated, the accuracy of the vena caval diameter changes to predict preload responsiveness is lower in spontaneously breathing patients. Accordingly, in healthy blood donors, it was not correlated with the cardiac output changes induced by blood removal [49]. The inferior vena cava diameter variation was found to be a poor predictor of fluid responsiveness in the emergency department where patients are usually spontaneously breathing [14, 50], although contradictory findings have been reported [51]. A recent study observed that, in patients with spontaneous breathing, only the respiratory variation of the inferior vena cava diameter of very high amplitude indicated fluid responsiveness [52].

Although the influence of tidal volume and lung compliance on the variation in vena caval dimensions have not been reported, it is likely that they limit the accuracy of vena caval diameter indices for assessing preload responsiveness, as with PPV and SVV. Nevertheless, in contrast to PPV and SVV, the vena caval diameter indices can be used in patients with cardiac arrhythmias (Fig. 2). It has also been demonstrated that the distensibility index of the right internal jugular vein was able to predict fluid responsiveness, even though the diagnostic accuracy was lower than reported for the inferior vena cava [53].

## Passive leg raising: the “internal” preload challenge

Passive leg raising (PLR) has been used for decades by rescuers as a first-line measure in patients with dizziness and syncope. Its interest in critical care has emerged after a study demonstrating that it induces significant changes in right and left cardiac preload [54]. A few years later, our group showed that PLR could be used as a reliable provocative test to detect preload responsiveness [55] (Fig. 1). The PLR test is in fact a reversible “preload challenge” of around 300 mL of blood [56] that can be repeated as frequently as required without infusing a drop of fluid. It has the advantage of being accurate in spontaneously breathing patients and with cardiac arrhythmias, low tidal volume ventilation and low lung compliance [36] (Fig. 2).

It has been recently shown that the infusion of blood induced by the postural change during a PLR is sufficient to induce a significant increase in the mean systemic pressure [57]. The resistance to venous return does not change, even in patients with intra-abdominal hypertension. In preload responders, the increase in mean systemic pressure is accompanied by an increase in the pressure gradient of venous return, in venous return itself and thus in cardiac output. By contrast in preload non-responders, the increase in the right atrial pressure that occurred simultaneously balanced the increase in mean systemic pressure, such that the pressure gradient of venous return (and thus cardiac output) remained unchanged [57].

Since 2006, many studies have confirmed the reliability of the PLR test with a remarkable consistency. Two meta-analyses of these studies have been recently published [58, 59]. In almost 1000 adult patients included in 21 studies, our team found that the pooled sensitivity was 85% and the pooled specificity was 91% [59]. The mean threshold that simultaneously provided the best sensitivity and specificity was a PLR-induced increase in cardiac output of 10% or more [59] (Table 1). The robust reliability of this test has likely contributed to its popularity and widespread application. The PLR test has been included in the last update of the recommendations of the Surviving Sepsis Campaign [60] and in a consensus conference of the European Society of Intensive Care Medicine [61].

A number of practical aspects regarding the technique of performing the PLR test are important to consider, as recently reviewed by Monnet and Teboul [62]. The most important is that the effects of the PLR must be assessed by the direct measurement of cardiac output (Table 1). It is important to recognise that changes in arterial pressure do not allow the assessment of the PLR haemodynamic effects with reliability; this has been confirmed by the recent meta-analyses [58, 59]. When the PLR-induced changes in arterial pulse pressure are used, the specificity remains very good, but the sensitivity of the test is much poorer. Moreover, cardiac output must be measured continuously and in real time. The haemodynamic effects of PLR reach their maximum within 1 min, diminishing rapidly thereafter in some patients, especially in patients with severe sepsis and capillary leak [63]. Intermittent measurements of cardiac output, like thermodilution, may thus be inappropriate.

Nevertheless, this does not imply that the PLR test necessarily requires invasive monitoring. Many studies have used non-invasive or minimally invasive techniques to estimate the PLR-induced changes in cardiac output [58, 59]. Both the calibrated and uncalibrated pulse contour analysis techniques are very convenient to use. Oesophageal Doppler, with measurement of the PLR effects on the aortic blood flow, was the first technique reported [55]. Echocardiography, with measurements of the PLR-induced changes in the velocity time integral of the left ventricular outflow tract, has been used in many studies. Even the PLR-induced changes in the peak velocity of the carotid [64] and femoral [65] arteries seem to be reliable indicators of the response of cardiac output to PLR. Bioreactance has been investigated with conflicting results [64, 66]. Endotracheal bioimpedance cardiography was reported to be unable to assess the haemodynamic response to a PLR test [67]. The totally non-invasive estimation of cardiac output by pulse contour analysis of the arterial curve obtained from photoplethysmography is also suitable for the PLR test, which may be particularly interesting out of the ICU and the operating room. The decrease in the pleth variability index during PLR has been shown to weakly detect the concomitant changes in cardiac output, especially with low specificity [68].

An original and totally non-invasive method is to measure the PLR-induced increase in end-tidal carbon dioxide (CO_2_) [69–71]. This technique requires that the patient has perfectly stable mechanical ventilation, in order to be sure that the changes in end-tidal CO_2_ are only related to changes in cardiac output. A recent study showed that the changes in end-tidal CO_2_ were able to detect the changes in cardiac output during PLR, but not during a mini-fluid challenge with 100 mL of saline [72].

It has been suggested that intra-abdominal hypertension invalidates the PLR test [73]. However, we believe that this message should be treated with caution. It is based on the hypothesis that the increased abdominal pressure induced by the PLR may compress the inferior vena cava and thereby interrupt the flow through it. However, this scenario has only been investigated in one study to date [74]. Moreover, the intra-abdominal pressure was not recorded during the PLR test in this study [74]. This is a significant flaw, as one could postulate that the PLR test could decrease the intra-abdominal hypertension by relieving the weight of the diaphragm on the abdominal cavity. Moreover, the PLR was observed to decrease, not increase, the resistance to venous return, in patients with intra-abdominal hypertension [57]. Additional studies are required to clarify this issue.

## End-expiratory occlusion test…

In patients undergoing mechanical ventilation, each insufflation decreases cardiac preload and tends to impede venous return. Interrupting mechanical ventilation for a few seconds stops this cyclic impediment in venous return. Cardiac preload transiently increases. If cardiac output increases in response to this end-expiratory occlusion (EEO) test, this indicates preload responsiveness of both ventricles (Fig. 1). Of note, the duration of the EEO must not be shorter than 15 s, likely because this lapse of time is required by the preload change to transit through the pulmonary circulation. Monnet et al. [75] observed that whether cardiac output measured by pulse contour analysis increased by more than 5% during the 15 s. EEO, a positive response to a subsequent fluid infusion, could be predicted with good sensitivity and specificity (Table 1).

The test has the advantage of being very easy to perform. It simply requires “stopping” the ventilator, as when measuring the intrinsic positive end-expiratory pressure, and measuring the changes in cardiac output. The EEO technique does not have the technical constraints of PLR. The EEO test is valid in patients with ARDS, a condition where PPV and SVV are not sensitive enough [36] (Fig. 2). In a study of patients with ARDS, the test remained valid at a PEEP level of 5 cmH_2_O as well as of 15 cmH_2_O [76]. The main limitation of the test is that it cannot be used in patients who are not intubated, and in patients who do not tolerate a 15-s respiratory hold (Table 2).

In published studies, the EEO test’s effects on cardiac output were assessed by pulse contour analysis [36, 75, 76]. This technique has the advantage of being very precise. Indeed, for the EEO test, the technique must be able to detect small changes in cardiac output. Pulse contour analysis performed on the arterial pressure curve obtained from photoplethysmography has likely the same capacity of assessing the effects of the EEO test. This might not be the case for the current version of bioreactance device, which averages cardiac output values on a too long time for being able to detect changes occurring in laps of a few seconds. The utility of using echocardiography has recently been investigated [77]. The increase in the velocity time integral of the left ventricular outflow tract during EEO was able to identify preload responsiveness, with a threshold of 4%. Interestingly in this study, the effects of an end-inspiratory occlusion were also assessed. A decrease in the velocity time integral of more than 5% during an end-inspiratory hold was able to detect preload-responsive patients. When both the effects of end-expiratory and end-inspiratory holds were added (in absolute values), they identified fluid responsiveness with a sensitivity or specificity that were not superior to either occlusion test taken separately, but with a threshold of 15%. This cut-off value is more compatible with the precision of echocardiography.

## … And other tests using heart–lung interactions

The idea of using heart–lung interactions to challenge preload responsiveness has led to other novel tests, less studied than the ones detailed above. In a recent study in patients undergoing cardiac surgery, fluid responsiveness was predicted by the haemodynamic effects of a sudden increase in positive end-expiratory pressure from 5 to 10 cmH_2_O. The effects were measured through CO_2_ elimination, which was used as a surrogate of cardiac output in these patients who were well sedated [78]. The respiratory systolic variation test (RSVT) quantifies the decrease in systolic pressure in response to a standardised manoeuvre consisting of three consecutive mechanical breaths with increasing airway pressure. The main advantage of RSVT is that it is independent of tidal volumes [79]. This test is now automatically performed by some ventilators, and the test appears to be as accurate as PPV and SVV [80].

## Fluid challenge: maxi or mini?

Infusing fluid is obviously the most direct way to challenge fluid responsiveness [81]. Nevertheless, the “conventional” fluid challenge has two major drawbacks. First, assessing its precise effects requires a direct measurement of cardiac output and cannot be based solely on the arterial pressure changes. In this regard, the fluid challenge has no advantage over the PLR test (Table 2). In a study where 500 mL saline was administered to critically ill patients, the changes in arterial pulse pressure only roughly detected the concomitant changes in cardiac output [82]. In particular, there were 22% of false negatives. In another study, there was no correlation between the changes in arterial pulse pressure and changes in cardiac output during a fluid challenge [83]. The discrepancy with the previous study [82] may be explained by the fact that the arterial pressure was measured at the radial artery and not the femoral site [83]. Nevertheless, both studies demonstrate that if one wants to precisely assess the effects of a fluid challenge, one must measure cardiac output and not rely on arterial pressure.

The second major drawback of a fluid challenge is that it is not a test but a treatment in its own right. In patients where multiple fluid challenges must be repeated in a short time, this inevitably leads to administering a volume of fluid that is far from negligible. For instance, in a patient with haemodynamic instability, where four or five episodes of hypotension occur in one day, performing fluid challenges will lead to infusing 2000 to 2500 mL of fluid that, by constitution, do not increase cardiac output. This obviously contributes to fluid overload and haemodilution with inherent risks of decrease in oxygen delivery to the tissues.

The idea has emerged to perform a fluid challenge with a volume of fluid much smaller than the “conventional” challenge. In a study where a “mini-fluid challenge” was performed with 100 mL of colloid, the changes in the velocity time integral of the left ventricular outflow tract measured with echocardiography predicted preload responsiveness [84]. The statistical threshold was a 6% increase in the velocity time integral. Nevertheless, since this threshold was below the precision of echocardiography, the authors suggested a 10% threshold, even though it reduced the test accuracy (Table 1). The main issue with the mini-fluid challenge is that small volumes of fluid can only induce small changes in cardiac preload and, in patients with preload responsiveness, only small changes in cardiac output. Thus, the test requires a very precise cardiac output monitoring system. Whether transthoracic echocardiography is precise enough is far from certain. It is even more doubtful if a 50-mL fluid challenge is used, as has been recently suggested [85]. By contrast, it is likely that the precision of non-invasive pulse contour analysis devices is enough for detecting the effects of a mini-fluid challenge. Whether it is the case also for bioreactance should be verified. The issue of precision is likely the reason why a study found that the mini-fluid challenge was not reliable when assessed by end-tidal CO_2_ [72]. Thus, although attractive, it is unclear that the mini-fluid challenge is reliable, especially when performed with techniques estimating cardiac output that are not very precise.

Recently, some authors sought to determine the smallest volume of fluid required to perform an effective fluid challenge by investigating the effects of different doses of intravenous fluids on changes in cardiac output (as measured by pulse contour analysis) and mean circulating filling pressure. In this study, a bolus of 4 mL/kg over 5 min was the smallest volume that could reliably increase the mean circulating filling pressure and make fluid challenge interpretable in every circumstance [86].

## Apply the concept of fluid responsiveness in a reasoned way!

Some important points must be kept in mind at the bedside. First, there are some instances where the tests we described in detail above are pointless because fluid responsiveness is obvious. In cases of haemorrhagic shock, clear-cut hypovolemic shock and the early phase of septic shock when fluid has not been administered, cardiac output will undoubtedly increase with fluid infusion. In such circumstances, delaying fluid administration is likely harmful, and therefore, tests of fluid responsiveness should not be used. Second, testing fluid responsiveness makes sense only in cases of circulatory failure (Fig. 2). The question of administering fluid or not can be asked only if cardiac output is to increase, i.e. in case of obvious or suspected tissue hypoxia [87]. In this regard, it must be kept in mind that preload responsiveness is a normal condition. Third, even if cardiac output increases, a positive test for fluid responsiveness should not automatically lead to fluid administration. In many instances, the risk of infusing fluid exceeds the expected benefit, and in each instance when a fluid bolus is contemplated, the risk benefit balance should be evaluated. For instance, in patients where acute circulatory failure and ARDS coexist, one should limit fluid administration even in cases of preload responsiveness because of the severity of lung injury [21], as assessed by increased lung water and by alteration of pulmonary vascular permeability [88]. The results of any of these tests should not be examined in isolation, but taking into account the entire picture of the patients. Moreover, one must keep in mind that none of these tests are 100% sensitive or specific. Every decision made from these tests must take this into account.

## Testing fluid responsiveness: not only for deciding to *administer* fluids

Testing for fluid responsiveness may help one to decide to administer fluid. However, equally important, testing for fluid responsiveness may help in the decision to stop fluid administration or not to administer fluids at all. The decision to stop fluid administration should be made on the disappearance of signs of circulatory failure, the appearance of signs of fluid overload and when the tests of preload responsiveness become negative [89]. Testing preload dependence may also be helpful in the de-escalation phase of shock management. At this stage, fluid removal is often undertaken, but the volume to be removed is difficult to estimate. In critically ill patients at the late phase of shock, our group recently showed that a PLR test performed before starting fluid removal predicts intradialytic hypotension with accuracy, especially with good specificity and positive predictive value [90]. This suggests that preload responsiveness should be assessed before starting fluid removal in order to avoid any haemodynamic deterioration.

## Conclusion

Because of the risk of fluid overload and the inconstant efficacy of volume expansion, the decision to administer fluid cannot be taken lightly. Fluids are drugs whose dose must be carefully titrated to the needs of the patient. Several methods and tests are currently available to identify preload responsiveness. All these techniques have some limitations (Table 2), but they are frequently complementary. The choice between the techniques for assessing fluid responsiveness depends on the patient’s condition and the available monitoring techniques (Fig. 2). It is important to stress that the decision of fluid administration should not be based solely on the presence of preload responsiveness, but also on the presence of haemodynamic instability (or peripheral hypoperfusion) and the absence of high risk for fluid overload. A reasoned fluid strategy estimating preload responsiveness to aid in the decision to administer fluid and to refrain from fluid administration will likely improve the quality of care delivered and patient outcomes.

## Abbreviations

CVP: central venous pressure
EEO: end-expiratory occlusion
ICU: intensive care unit
PLR: passive leg raising
PPV: pulse pressure variation
SVV: stroke volume variation
